# Effect of premature luteinizing hormone surge on pregnancy outcomes in intracytoplasmic sperm injection or *in vitro* fertilization cycles

**DOI:** 10.3389/fmed.2024.1429033

**Published:** 2025-03-21

**Authors:** Maram Aldarsooni, Mohammad Alfarah, Fatimah Albohammod, Dania Al-Jaroudi

**Affiliations:** ^1^Obstetrics and Gynecology Department, Women's Specialized Hospital, King Fahad Medical City, Riyadh, Saudi Arabia; ^2^Obstetrics and Gynecology Department, Specialized Medical Center Hospital, Riyadh, Saudi Arabia; ^3^Reproductive Endocrine and Infertility Medicine Department, King Fahad Medical City, Riyadh, Saudi Arabia

**Keywords:** LH surge, Cetrorelix, pregnancy, *in-vitro* fertilization cycles, intracytoplasmic sperm injection

## Abstract

**Background:**

A premature surge in luteinizing hormone (LH) during ovarian stimulation cycles between 12 and 25% has been commonly reported. However, there is still a lack of consensus on whether premature LH surge affects pregnancy outcomes.

**Objective:**

This study aimed to evaluate the effect of premature LH surge on pregnancy rates in women with and without premature LH surge during intracytoplasmic sperm injection or stimulation of *in vitro* fertilization cycles at a tertiary hospital in Riyadh, Saudi Arabia.

**Materials and methods:**

A cross-sectional study was conducted. Information related to 771 women who had IVF/ICSI cycles between January 2022 and December 2022 was reviewed. Patients were divided into two groups based on premature LH surge.

**Results:**

There was no significant difference in patients’ characteristics. The live birth rates were significantly higher in women without premature LH surge than in women with LH surge (12.9% vs. 5.6%) (*p* = 0.010). With regard to the number of oocytes collected, M2, and fertilization rate, these were significantly higher in women without premature LH surge, with *p-*values of 0.001, 0.002, and 0.004, respectively.

**Conclusion:**

The study demonstrates significantly higher live birth rates in women without premature LH surge.

## 1 Introduction

Luteinizing hormone (LH) and follicle-stimulating hormone (FSH) are gonadotropins secreted from the anterior pituitary gland and play an important role during folliculogenesis (1, 2). FSH binds to the granulose cells to stimulate follicle growth, while LH acts on the theca cells to produce and secrete androgen (1). During the course of a natural cycle, a high concentration of FSH in the early follicular phase initiates follicular development, and with the growth of this follicle, an increase in the level of serum estradiol (E2) occurs (3). The rise in estrogen levels causes negative feedback on the pituitary gland, leading to a reduction in the FSH levels, which results in the survival of only one dominant follicle. At the same time, atresia occurs in the other small follicles. After that, the estrogen levels continue to increase and become high enough to cause positive feedback on the pituitary gland; however, the effect currently reflects only on the LH and a surge occurs in the LH. As a result of this surge, final maturation of the oocyte takes place, leading to ovulation.

During ovulation induction, the administration of exogenous gonadotropins leads to the growth of multiple follicles, resulting in an increase in serum estradiol compared to the natural cycle. The trigger estradiol concentration for pre-ovulatory LH surge occurs before the complete development of the follicles. Thus, it is important to prevent premature LH surge and ovulation for the success of the assisted reproductive treatment (3).

Previous studies have reported that a premature LH surge leads to adverse outcomes, including higher cycle cancelation, decreased fertilization rate, embryo quality, implantation rate, and pregnancy rate (1, 4). Cassidenti et al. reported the first successful administration of a gonadotropin-releasing hormone (GnRH) antagonist to prevent premature LH surge during the *in vitro* fertilization (IVF) cycle (5). However, when premature LH surge occurred, no clear cutoff was reported for serum LH, indicating cancelation of the IVF cycle (6).

Another definition is an LH level >10 IU/L or >50% increase from the baseline during ovarian stimulation. A premature rise in LH level may result in a reduced ongoing pregnancy rate in fresh embryo transfer cycles. However, a better understanding might be yielded when there is a comparison between fresh and frozen embryo transfers with a premature rise in LH (1, 7).

In the ovarian stimulation cycle, premature LH surge can reach as high as 12% (1) and has been reported up to 25% by another study. As many as 1.2% of these cycles are canceled due to premature LH surge despite the administration of GnRH antagonists to prevent it (5, 6). With regard to earlier ovum pick-up in women with premature LH surge, Choi et al. reported that oocytes retrieved within 36 h from the day of HCG administration were ineffective in preventing cancelation, with no difference in the number of oocytes retrieved. However, despite the low fertilization rate in women with oocytes retrieved within 36 h compared to women after 36 h, it was observed that the pregnancy rate was higher in those with earlier retrieval of oocytes (6).

There is still a lack of consensus on whether premature LH surge affects pregnancy outcomes in fresh embryo transfer cycles. According to some studies, a premature LH surge has been linked to a decrease in clinical pregnancy rates (7–9), whereas others reported no adverse impact on pregnancy rates (10).

Therefore, this study aimed to compare the clinical outcomes, particularly pregnancy rates in women with premature LH surge and those without premature LH surge when undergoing intracytoplasmic sperm injection (ICSI) or IVF cycles at King Fahad Medical City (KFMC), Riyadh, Saudi Arabia.

## 2 Materials and methods

This single-center, retrospective comparative study was conducted from January to December 2022 at the Reproductive, Endocrinology and Infertility Center, Women Specialized Hospital in KFMC.

The inclusion criteria specified that participants were women with primary or secondary infertility, aged between 21 and 42 years, nulliparous, and underwent IVF/ICSI cycles at KFMC using antagonist protocol (either fixed or flexible). Women over 42 years old or those who underwent IVF/ICSI cycles using a long protocol were excluded.

A total of 771 patients who fit the inclusion criteria were included in the study and were divided into two groups based on premature LH surge. Group A (*n* = 36) consisted of patients who experienced a premature LH surge, while Group B (*n* = 735) included those who did not. A premature LH surge was defined as an LH level of ≥10 IU/L or three times the baseline LH level.

### 2.1 Stimulation protocol

FSH, LH, and E2 were measured on the second, third, or fourth day of the cycle. LH is measured two to three times per cycle and on the day of the trigger. A pelvic ultrasound was performed on the same day to exclude any abnormalities. Each patient underwent stimulation using (either fixed on day 5 or day 6 of the cycle or flexible when the size of the follicle is ≥12 mm) GnRH antagonist protocol. The patient received either single or multiple gonadotropin that was dose adjusted according to the follicular development and E2 levels. Cetrorelix (0.25 mg) was administrated when the E2 levels reached ≥500 pmol/L or when the size of the follicles became ≥12 mm. When the dominant follicles became ≥18–20 mm, recombinant HCG was administrated. Oocyte collection was performed via transvaginal ultrasound guidance 36 h after the HCG trigger. Luteal support was initiated with Cyclogest^®^ (LDCollins, UK) vaginal progesterone suppositories at a dose of 400 mg, starting the day after the collection of oocytes. Embryo transfer was performed between days 3 and 5 after the collection of oocytes. Cancelation of fresh embryo transfer was performed for the following reasons: to avoid the risk of ovarian hyperstimulation syndrome, in cases of no fertilization, or if no oocytes were collected.

A biochemical pregnancy was defined as a *β*-HCG level of 50 IU/L 14 days after the embryo transfer. An abortion was defined as pregnancy loss at ≤20 weeks of gestation. Preterm live birth was defined as the delivery of a live baby between 20 weeks and 37 weeks, while term live birth was defined as the delivery of a live baby at 37 weeks.

### 2.2 Data collection

After ethical approval was obtained, data collection sheets were distributed among co-investigators of the project. The data on IVF/ICSI cycles that were carried out during the study period were collected from the records of the Reproductive and Infertility Department through the EPIC and HIM databases as well as the Embryology Lab database. The data collected included age, type of infertility, trial number, Cetrorelix administration day, type and total dose of medications, stimulation duration, basal levels of FSH and LH, estradiol levels, LH and estradiol levels on trigger day, number of oocytes retrieved, injected, and fertilized, fresh embryo transfers and frozen embryo transfers, quality of eggs, pregnancy outcomes, ectopic pregnancies, and live births.

### 2.3 Ethical considerations

This study was approved by the Ethics Committee of KFMC, Riyadh, Saudi Arabia (IRB No: 23-569).

### 2.4 Statistical analysis

All categorical variables, such as type of infertility, trial group, medications, day of Cetrorelix administration, LH surge, and frozen embryo transfers, were presented as frequency and percentage. Continuous variables, such as age, total dose, stimulation duration, basal levels of FSH, and basal levels of LH were expressed as median [interquartile range (IQR)]. The Kolmogorov–Smirnov test was used to confirm the assumption of normal distribution. If the data were not normal, a non-parametric test was used. Pearson’s chi-square/Fisher’s exact test was used to determine significant associations between categorical variables, depending on whether the cell was expected to have an expected frequency of less than 5. The Mann–Whitney *U*-test was applied to determine the significance between continuous data and LH surge and no LH surge. A two-sided *p*-value less than 0.05 was considered statistically significant. All data were entered and analyzed using the SPSS 25 Statistics Package (SPSS Inc., Chicago, Illinois, United States).

## 3 Results

A total of 771 women were included in our study and were divided into two groups: Group A (*n* = 36, 4.6%) consisted of women who had premature LH surge, and Group B (*n* = 735, 95.3%) consisted of women who did not have premature LH surge; 125 cycles had no oocytes retrieved (16.2%).

Table 1 displays the demographic and clinical characteristics of the patients. The majority of the patients (*n* = 717, 93%) were diagnosed with primary infertility, and the median age for the patients was 34 years. The trial number ≤ 3 was for the majority 688 (89.2%); 38.7% of the patients received a combination of Gonal F and Menopur, while 23.7% received Gonal F and Luveris. The majority of patients, 688 (89.2%) had less or equal to three previous IVF/ICSI cycles. In terms of the reasons for no fresh ETs, 8.9% of the patients experienced freeze-all or ovarian hyperstimulation syndrome, and 5.4% had no fertilization. In contrast, 77.8% underwent fresh embryo transfers. Among the patients, 68.4% (*n* = 527) had zero frozen embryos available. Regarding the quality of eggs, 484 patients (62.8%) had >50% good quality. A negative pregnancy test was observed in half of the patients (52.0%).

**Table 1:** Demographic and clinical characteristics of patients (*n* = 771).

Variables	Description	*n* (*n*%)
Type of infertility	Primary	717 (93.0%)
	Secondary	54 (7.0%)
Age (years)	Median [IQR]	34 [38–30]
Trial number	≤3	688 (89.2%)
	>3	83 (10.8%)
Medications	Gonal F	136 (17.6%)
	Merional	130 (16.9%)
	Menopur	24 (3.1%)
	Gonal F and Merional	0 (0.0%)
	Gonal F and Luveris	183 (23.7%)
	Gonal F and Menopur	298 (38.7%)
GnRH antagonist, Cetrorelix start day	Day 5–6	653 (84.7%)
	Others	118 (15.3%)
Total dose	Median [IQR]	3,000 [4,500–1,975]
Stimulation duration	Median [IQR]	10 [12–9]
Basal FSH	Median [IQR]	5.95 [7.45–4.88]
Basal LH	Median [IQR]	3.8 [5–2.7]
Basal E2	Median [IQR]	155 [192–123]
E2 at trigger	Median [IQR]	4,772 [8,387–2,402]
LH at trigger	Median [IQR]	1.5 [2.6–0.9]
Oocyte retrieved	Median [IQR]	7 [10–4]
Injected	Median [IQR]	4 [8–3]
Fertilization	Median [IQR]	3 [5–1]
Fresh embryo transfer	Median [IQR]	1 [2–1]
LH surge	Yes	36 (4.7%)
	No	735 (95.3%)
Cause of no fresh embryo transfer	Freeze all or OHSS	69 (8.9%)
	No fertilization	42 (5.4%)
	Others	60 (7.8%)
	N/A (fresh embryo transfer done)	600 (77.8%)
Frozen embryo	0 frozen embryo	527 (68.4%)
	1 frozen embryo	23 (3.0%)
	2 frozen embryo	74 (9.6%)
	3 frozen embryo	55 (7.1%)
	>3 frozen embryo	92 (11.9%)
Quality of eggs	>50% good	484 (62.8%)
	<50% good	162 (21.0%)
	N/A	125 (16.2%)
Outcomes	Biochemical	20 (2.6%)
	Abortion	68 (8.8%)
	Ectopic	0 (0.0%)
	Live birth (term/preterm)	97 (12.6%)
	N/A	178 (23.1%)
	Negative	401 (52.0%)
	Unknown	7 (0.9%)

Categorical data are presented as frequency (%) while continuous data are expressed as median [IQR: interquartile range].

As seen in Table 2, no significant difference was found with respect to age, type of infertility, total dose of stimulation medications, and duration and day of Cetrorelix administration. The median age of the women in Group A and Group B was 36.5 and 34 years, respectively, which was not found to be statistically significant (*p* = 0.066). With regard to the basal hormonal levels between the two groups, there was no significant difference except for the basal E2 levels, which were higher in group B (*p* = 0.033). The LH levels on trigger day were significantly higher in group A (*p* ≤ 0.001). The number of oocytes collected, oocytes injected, total fertilization rates, and quality of the eggs were significantly lower in group A (*p* = 0.001, *p* = 0.002, *p* = 0.004, and *p* = 0.015, respectively). The clinical results showed that chemical and biochemical pregnancy rates were higher in Group A at 2.8% than in Group B (2.6%). The total number of pregnancies among women with a premature LH surge was 3 (8.4%), compared to 182 (24.8%) in those without a premature LH surge. Furthermore, the number of live births in group A was 2 (5.6%) compared to 95 (12.9%) in group B.

**Table 2:** Impact and association between premature LH surge and IVF/ICSI cycles parameters.

Variables	Description	LH surge		*p*-value
		Yes (*n* = 36)	No (*n* = 735)	
Type of infertility	Primary	32 (88.9%)	685 (93.2%)	0.323
	Secondary	4 (11.1%)	50 (6.8%)	
Age	Median [IQR]	36.5 [40–31.25]	34 [38–30]	0.066
Trial number	≤3	33 (91.7%)	655 (89.1%)	0.630
	>3	3 (8.3%)	80 (10.9%)	
Medications	rFSH (Gonal f)	9 (25.0%)	127 (17.3%)	0.209
	hMG (Merional)	4 (11.1%)	126 (17.1%)	
	hMG (Menopur)	0 (0.0%)	24 (3.3%)	
	rFSH (Gonal f, LH (luveris)	5 (13.9%)	178 (24.2%)	
	rFSH (gonal f) and hMG (Menopur)	18 (50.0%)	280 (38.1%)	
GnRH antagonist, Cetrorelix day	Days 5–6	27 (75.0%)	626 (85.2%)	0.098
	Others	9 (25.0%)	109 (14.8%)	
Total dose	Median [IQR]	3,675 [5962.5–1884.38]	3,000 [4,500–1987.5]	0.137
Stimulation duration	Median [IQR]	10 [12.75–9]	10 [12–9]	0.870
Basal FSH	Median [IQR]	6.5 [8.68–4.84]	5.9 [7.43–4.88]	0.201
Basal LH	Median [IQR]	3.2 [6.53–2.3]	3.8 [5–2.7]	0.859
Basal E2	Median [IQR]	181 [219.5–139]	154 [191–12]	*0.033
E2 at trigger	Median [IQR]	2,654 [9263.5–801.25]	4,861 [8,336–2,441]	0.052
LH at trigger	Median [IQR]	3.15 [8–1.33]	1.5 [2.5–0.9]	*<0.001
Oocyte retrieved	Median [IQR]	3.5 [7.75–1]	7 [10–4]	*0.001
Injected	Median [IQR]	2 [5.75–1]	5 [8–3]	*0.002
Fertilization	Median [IQR]	1 [3.75–0]	3 [5–1]	*0.004
Cause of no fresh ET	Freeze all or OHSS	7 (19.4%)	62 (8.4%)	*0.009
	No fertilization	3 (8.3%)	39 (5.3%)	
	others	6 (16.7%)	54 (7.3%)	
	Fresh embryo transfer done	20 (55.6%)	580 (78.9%)	
Quality of eggs	>50% good	19 (52.8%)	465 (63.3%)	*0.015
	<50% good	5 (13.9%)	157 (21.4%)	
	N/A	12 (33.3%)	113 (15.4%)	
Outcomes	Biochemical pregnancy	1 (2.8%)	19 (2.6%)	*0.010
	Abortion	0 (0.0%)	68 (9.3%)	
	Ectopic pregnancy	0 (0.0%)	0 (0.0%)	
	Pregnant	3 (8.4%)	182 (24.8%)	
	Live birth (term/preterm)	2 (5.6%)	95 (12.9%)	
	N/A	17 (47.2%)	161 (21.9%)	
	Negative	16 (44.4%)	385 (52.4%)	
	Unknown	0 (0.0%)	7 (1.0%)	

Categorical data are presented as frequency (%) while continuous data are expressed as median [IQR: interquartile range]; *shows that the *p*-value is significant at *p* < 0.05.

## 4 Discussion

This study aimed to evaluate the effect of premature LH surge on pregnancy rate in comparison with a group of women without premature LH surge. Our study showed that the incidence of premature LH surge was 4.6%.

In our study, the fixed protocol GnRH antagonist was started on days 5–6 of stimulation, while in the flexible protocol, the GnRH antagonist was started when E2 ≥ 500–600 or when a dominant follicle size of ≥12 mm was reached. A total of 653 women in our study underwent fixed protocol, with 4.1% of them experiencing a premature LH surge. Meanwhile, 118 women underwent flexible protocol and 7.6% of them experienced a premature LH surge. Thus, our findings suggest that the occurrence of premature LH surge was more likely in the flexible group, and this result is in line with other studies (6).

In the present study, the pregnancy rates among women with and without a premature LH surge were 8.4 and 24.8%, respectively, while the live birth rates were 5.6 and 12.9%, respectively. Both the pregnancy rate and live birth rate were significantly higher in women without premature LH surge (*p* = 0.010). Many studies showed that premature LH surge can be avoided by administration of exogenous GnRH antagonists (1, 4), regardless of the stimulation protocol whether fixed or flexible GnRH antagonist; however, it is more effective with a flexible protocol (6). Sönmezer et al. reported that an immediate decrease in LH surge was observed after GnRH antagonist administration, dropping from very high levels of 36 and 47 IU/mL to less than 10 IU/mL (4).

Gao et al. also reported lower cumulative live birth rates in patients (≥37 years) with premature LH surge than those without it (β: 0.20; 95% CI: 0.05–0.88; *p* = 0.03) (11). In their meta-analysis of fixed versus flexible protocol, Al-Inany et al. reported a trend toward a higher pregnancy rate among women treated with the fixed protocol (12). Thus, it can be presumed that the high pregnancy rate observed in the fixed protocol may be related to a lower incidence of premature LH surge among patients on the fixed protocol.

Many studies reported no significant difference in pregnancy outcomes in women with and without premature LH surge once GnRH antagonist was used (13, 14). In contrast to a study by Zhang et al., our study found that the numbers of oocytes retrieved, M2, and fertilization rate were higher in women without the premature LH surge group than with the premature LH surge group (1). However, our result for the number of oocytes retrieved, mature oocytes, and fertilization rate was similar to the result observed in a study conducted by Frattarelli et al. (3).

We recognize the limitations of this study, including its single-centered and retrospective nature, as well as the small sample size of the patients with premature LH surge. Therefore, further studies with a larger sample size are needed. Nevertheless, prevention of LH surge has been shown to control the function of granulosa cells, leading to its growth, thus enabling normal follicle and oocyte development. Therefore, LH surge prevention yields more embryos, higher pregnancy rates, and higher cumulative live birth rates.

Another limitation is that the cause–effect relationship was discussed only concerning antagonist protocols. Control for confounding variables should have been considered to clarify observed relationships.

Linear regression could be employed to explore factors associated with the occurrence of a premature LH surge, while logistic regression is necessary for assessing variables related to LH rise that predict the main outcome (live birth rate). However, these analyses were not conducted. Another limitation is the lack of data on the cumulative live birth rate (LBR) between the two groups.

## 5 Conclusion

In conclusion, the present study suggests live births were significantly higher in women without premature LH surge during IVF/ICSI cycles. Moreover, the number of oocytes collected, M2, and fertilization rate were also found to be significantly higher in women without premature LH surge.

## Data Availability

The original contributions presented in the study are included in the article/supplementary material, further inquiries can be directed to the corresponding author.

## References

[1] Zhang Y Xu Y Yu J Wang X Xue Q Shang J. Effect of a premature luteinizing hormone surge on cumulative live birth rate during a flexible GnRH antagonist protocol: a retrospective study. preprint version 1: Research Square (2022). doi: 10.21203/rs.3.rs-1361146/v1, PMC10294483 37370146

[2] Zhang W Liu Z Liu M Li J Guan Y. Is it necessary to monitor the serum luteinizing hormone (LH) concentration on the human chorionic gonadotropin (HCG) day among young women during the follicular-phase long protocol? A retrospective cohort study. Reprod Biol Endocrinol. (2022) 20:24. doi: 10.1186/s12958-022-00888-4, PMID: 35105359 PMC8808976

[3] Frattarelli JL Hillensjö T Broekmans FJ Witjes H Elbers J Gordon K. Clinical impact of LH rises prior to and during ganirelix treatment started on day 5 or on day 6 of ovarian stimulation. Reprod Biol Endocrinol. (2013) 11:90. doi: 10.1186/1477-7827-11-90, PMID: 24028076 PMC3847921

[4] Sönmezer M Pelin Cil A Atabekoğlu C Ozkavukçu S Ozmen B. Does premature luteinization or early surge of LH impair cycle outcome? Report of two successful outcomes. J Assist Reprod Genet. (2009) 26:159–63. doi: 10.1007/s10815-009-9299-5, PMID: 19224360 PMC2654938

[5] Cassidenti DL Sauer MV Paulson RJ Ditkoff EC Rivier J Yen SS. Comparison of intermittent and continuous use of a gonadotropin-releasing hormone antagonist (Nal-Glu) in *in vitro* fertilization cycles: a preliminary report. Am J Obstet Gynecol. (1991) 165:1806–10. doi: 10.1016/0002-9378(91)90036-Q 1750479

[6] Choi MH Cha SH Park CW Kim JY Yang KM Song IO. The effectiveness of earlier oocyte retrieval in the case of a premature luteinizing hormone surge on hCG day in *in vitro* fertilization-embryo transfer cycles. Clin Exp Reprod Med. (2013) 40:90–4. doi: 10.5653/cerm.2013.40.2.90, PMID: 23875165 PMC3714434

[7] Dovey S McIntyre K Jacobson D Catov J Wakim A. Is a premature rise in luteinizing hormone in the absence of increased progesterone levels detrimental to pregnancy outcome in GnRH antagonist *in vitro* fertilization cycles. Fertil Steril. (2011) 96:585–9. doi: 10.1016/j.fertnstert.2011.06.042, PMID: 21774931

[8] Geng Y Lai Q Xun Y Jin L. The effect of premature luteinizing hormone increases among high ovarian responders undergoing a gonadotropin-releasing hormone antagonist ovarian stimulation protocol. Int J Gynaecol Obstet. (2018) 142:97–103. doi: 10.1002/ijgo.12485, PMID: 29542120

[9] Zhang D Zhang D Sun Z Deng C Yu Q Zhen J. The effect of a transient premature luteinizing hormone surge without elevated serum progesterone on *in vitro* fertilization outcomes in a gonadotropin-releasing hormone antagonist flexible protocol. Gynecol Endocrinol. (2020) 36:550–3. doi: 10.1080/09513590.2019.1683730, PMID: 31829082

[10] Kummer NE Weitzman VN Benadiva CA Schmidt DW Engmann LL Nulsen JC. *In vitro* fertilization outcomes in patients experiencing a premature rise in luteinizing hormone during a gonadotropin-releasing hormone antagonist cycle. Fertil Steril. (2011) 95:2592–4. doi: 10.1016/j.fertnstert.2010.12.046 21292260

[11] Gao F Wang Y Wu D Fu M Zhang Q Ren Y. A premature rise of luteinizing hormone is associated with a reduced cumulative live birth rate in patients ≥37 years old undergoing GnRH antagonist *in vitro* fertilization cycles. Front Endocrinol (Lausanne). (2021) 12:722655. doi: 10.3389/fendo.2021.722655, PMID: 34925227 PMC8678590

[12] Al-Inany H Aboulghar MA Mansour RT Serour GI. Optimizing GnRH antagonist administration: meta-analysis of fixed versus flexible protocol. Reprod Biomed Online. (2005) 10:567–70. doi: 10.1016/S1472-6483(10)61661-6, PMID: 15949209

[13] Wilcox J Potter D Moore M Ferrande L Kelly E. Prospective, randomized trial comparing cetrorelix acetate and ganirelix acetate in a programmed, flexible protocol for premature luteinizing hormone surge prevention in assisted reproductive technologies. Fertil Steril. (2005) 84:108–17. doi: 10.1016/j.fertnstert.2005.03.016 16009165

[14] Zhang J Zhou X Chen Y Zhang Q Li Y Zhe J. Effects of cetrorelix versus ganirelix in gonadotropin-releasing hormone antagonist cycles for preventing premature luteinizing hormone surges and on clinical outcomes of IVF-ET cycles. Nan Fang Yi Ke Da Xue Xue Bao. (2019) 39:1207–12. doi: 10.12122/j.issn.1673-4254.2019.10.12, PMID: 31801718 PMC6867944

